# Bent Metal in a Bone: A Rare Complication of an Emergent Procedure or a Deficiency in Skill Set?

**DOI:** 10.1155/2016/4382481

**Published:** 2016-11-27

**Authors:** Mridula Krishnan, Katherine Lester, Amber Johnson, Kaye Bardeloza, Peter Edemekong, Ilya Berim

**Affiliations:** ^1^Department of Internal Medicine, CHI Creighton University Medical Center, Omaha, NE, USA; ^2^Creighton University School of Medicine, Omaha, NE, USA; ^3^Department of Family Medicine, CHI Creighton University Medical Center, Omaha, NE, USA; ^4^Department of Pulmonary and Critical Care, CHI Creighton University Medical Center, Omaha, NE, USA

## Abstract

Intraosseous (IO) access is an important consideration in patients with difficult intravenous (IV) access in emergent situations. IO access in adults has become more popular due to the ease of placement and high success rates. The most common sites of access include the proximal tibia and the humeral head. The complications associated are rare but can be catastrophic: subsequent amputation of a limb has been described in the literature. We report a 25-year-old female presenting with diabetic ketoacidosis (DKA) in whom emergent IO access was complicated by needle bending inside the humerus. Conventional bedside removal was impossible and required surgical intervention in operating room.

## 1. Introduction

Intraosseous (IO) access can be lifesaving when peripheral vascular access is difficult to obtain and the complications are minimal [1, 2]. Its use is more commonly observed in the pediatric subset of patients due to the ease of access but adult IO placement is becoming a more frequent practice with high success rates [3].

Efficacy of medication administration via intravenous (IV) versus intraosseous (IO) route has been found to be comparable in onset and duration of action of pharmacological agents [1]. We report a case of a 25-year-old female who required placement of an IO needle with the EZ-IO system for treatment of severe dehydration and hemodynamic instability as a complication of diabetic ketoacidosis (DKA).

## 2. Case Presentation

A 25-year-old female presented to the emergency department with complaints of severe nonradiating epigastric and umbilical pain associated with nausea and vomiting. She was unable to tolerate oral intake. The patient reported this pain to be similar to the abdominal pain that occurred with previous episodes of DKA, although more severe in intensity. Past medical history was significant for type 1 diabetes mellitus with reported noncompliance with insulin and multiple episodes of DKA. Medical history also included asthma, bipolar disorder, ischemic bowel disease status after small bowel resection, methamphetamine abuse, posttraumatic stress disorder, and idiopathic chronic pancreatitis. Her home medications comprised of a long and short acting daily insulin regimen, divalproex, citalopram, and albuterol inhaler. She had no documented allergies.

Vital signs on arrival revealed a heart rate of 115/minute, blood pressure of 132/110 mmHg, respiratory rate of 28/minute, and oxygen saturation of 100% on room air. On physical exam, the patient was noted to be lethargic with dry mucous membranes. Cardiovascular examination revealed sinus tachycardia. Abdominal examination revealed diffuse abdominal tenderness with active bowel sounds with no evidence of guarding or rigidity.

Due to the severity and acuity of her uncontrolled diabetic ketoacidosis with difficulty obtaining IV access, an intraosseous line was obtained in the right humerus for administration of intravenous fluids. A registered nurse, with prior IO access training that included a class and further instruction at hospital orientation when hired, obtained IO access using the EZ-IO system. Initial laboratory studies revealed a blood glucose level of 321 mg/dL, an anion gap of 22, and bicarbonate level of 15 mmol/L. The potassium level was 3.6 without electrocardiographic changes. Urine analysis was positive for ketones and glucose. Arterial blood gas revealed severe metabolic acidosis with the pH being 7.05. Abdominal radiograph was unremarkable. The patient was diagnosed with severe diabetic ketoacidosis and aggressive fluid resuscitation was initiated with normal saline through the intraosseous access, along with insulin infusion as per the hospital's DKA protocol. There were no difficulties with fluid and medication administration through the aforementioned intraosseous needle.

**Figure 1:**
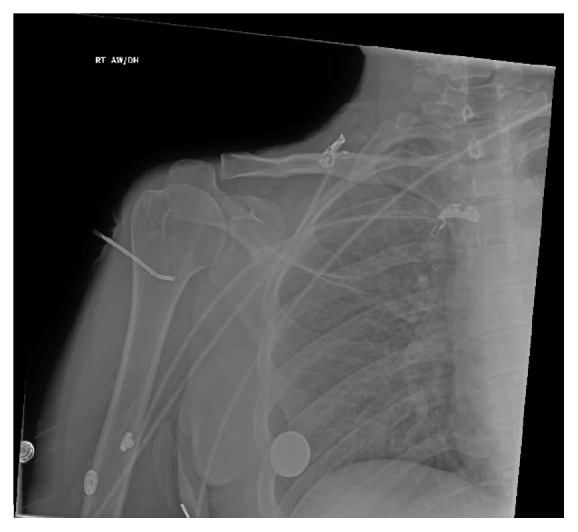
Radiograph of the right shoulder with a bent intraosseous needle in the neck of the humerus.

After adequate fluid resuscitation, an attempt at intraosseous line removal in the intensive care unit was unsuccessful due to severe pain in addition to concerns for possible breakage of the needle. A plain radiograph of the right shoulder was significant for an intraosseous needle that appeared bent at the humeral neck, without any evidence of fracture or dislocation on anteroposterior view (Figure 1).

**Figure 2:**
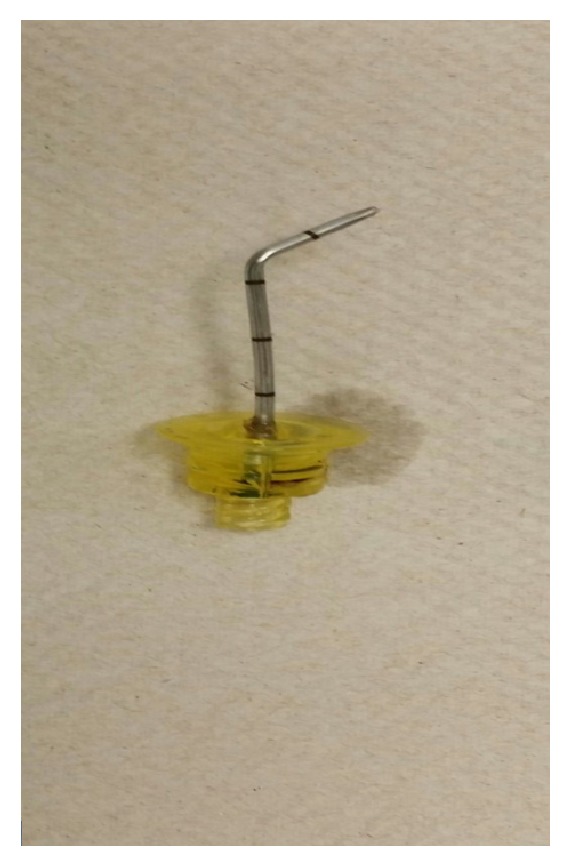
Bent EZ-IO after surgical removal from the bone.

Orthopedics was consulted and the risks and benefits of surgical and nonsurgical options for intraosseous line removal were thoroughly discussed with the patient. The patient opted to undergo surgical removal of the intraosseous line. Intraoperatively, the right arm was abducted and under C-arm guidance, gentle traction on the intraosseous line was placed directly over the bent portion in order to prevent the needle from breaking off inside the bone. The needle was removed in one piece and C-arm images were taken to confirm no pieces of needle were left behind (Figure 2). Intraoperative fluoroscopic images demonstrated removal of the intraosseous needle from the proximal humerus, with no evidence of residual foreign body.

Upon further investigation, the nurse reported two prior successful tibial IO placements. However, the nurse denied having placed a humeral IO line prior to this patient interaction. The nurse noted that the patient refused a stabilizing device to keep her arm stable during the placement. The patient had a history of bent intraosseous needles when removing the needles in the past. The prior incidents did not require surgery to remove the intraosseous needle. After the event, the emergency department nurses received training in obtaining IV access via humeral and tibial IO placement.

## 3. Discussion

IO infusions are a means of achieving rapid administration of medications into the intravascular compartment in emergency situations [8]. American Heart Association (AHA) and European Resuscitation Council (ERC) both recommend IO access if IV access cannot be obtained especially in emergent situations [9, 10]. IV access failure rates in the emergency department have been reported to be between 10 and 40 percent [11]. The time required to obtain peripheral IV access averages between 2 and 16 minutes in those with difficult peripheral vascular access [6, 11].

There have been multiple large prospective studies based on pediatric literature to assess the safety and efficacy of an intraosseous line placement. The use of semiautomatic IO (EZ-IO) has led to increased use of IOs to obtain peripheral access [12].

In our patient, it was difficult to determine a single event that led to this complication. The various factors that could have contributed to the bend in the needle include patient's inability to maintain appropriate arm position during procedure, level of nurse experience with IO access, incorrect site of placement of the IO, manipulation during removal, improper positioning of the upper limb during and after IO placement, or a defect in manufacturing of the IO needle. There should be major emphasis on correct positioning of the IO needle and also the prevention of dislodgement to prevent such complications. The needle is inserted into the skin perpendicular to the bone and once the needle penetrates the bone marrow cavity, a loss of resistance is detected. When using a power-driven EZ-IO device, the drill has to be stopped within a certain distance so that the needle will remain in the IO space and not penetrate the opposite cortex. In our case, besides the above factors that could have predisposed to the event, it can also be speculated that incorrect size of the needle was used.

Below we will discuss intraosseous access in detail with a focus on the complications of the technique.

### 3.1. Types of IO

There are many commercially available intraosseous devices [13]. The ones approved by the Food and Drug Administration (FDA) include the First Access for Shock and Trauma 1 (FAST1), the EZ-IO, and the Bone Injection Gun (BIG). The EZ-IO is a battery operated drill which is most frequently used [2, 13, 14].

The FAST1 is a spring device which was specially designed to obtain IO access through the sternum [14, 15].

### 3.2. Sites of IO Access

The sternum was used for IO infusions previously; however, the tibia and humerus have been found to be more advantageous [16]. The tibia and humerus are both long bones and easy to palpate and have easily identifiable landmarks. A nonrandomized, prospective, observational study by Ong et al. compared the infusion rates, rates of successful placement, time to placement, and complications for tibial or humeral IO access using the EZ-IO device. The results indicated no significant difference in flow rates between the two placements. In addition, there were no significant differences in the complication rates between the two different access sites. Advantage of gaining tibial access includes easily palpable and identifiable landmarks [17]. On the other hand, while the aforementioned study found no difference in infusion rates, a cadaveric study by Pasley et al. found advantages to humeral placement to include capability for faster infusion rates and possible decreased time to central circulation. Flow rate in the humerus was found to be greater than in the tibia, with average flow rate of 57.1 mL/min at the humerus and average flow rate of 30.7 mL/min at the proximal tibia [18]. These findings are consistent with previous trials in swine models [19, 20]. Randomized controlled trials comparing time to central circulation have not yet been conducted, but internal report by producers of the EZ-IO suggests that time from injection at humerus insertion site to entry into the superior vena cava is only 2.3 seconds, [21] which may indicate a second advantage of humeral placement.

### 3.3. Complications Associated with IO Access

 Barlow and Kuhn analyzed the complication rate with the use of IO catheters in a large subset of over 5000 patients and the overall complication rate was as low as 2.1 percent [22].

A bend in the intraosseous line was more commonly observed in manually inserted IOs rather than in cases with the use of a drill-set [5, 23]. Using a live swine model, a study comparing manually placed versus mechanical drill-assisted IO catheters reported 33.3 percent bent needles via manual insertion which made intraosseous infusion impossible. However, no bent needles were reported using mechanical drill-assistance [5]. A study by Brenner et al. reported that 15.4 percent of the time establishing IO access manually resulted in complications such as a bent or broken insertion needle [23]. A Scandinavian study reported bent needles in 4 percent of the patients following insertion of an IO. The most common presenting signs of bent IO needle in these cases were difficulty in penetration of the periosteum and difficult bone marrow aspiration following insertion [2]. The bent needles caused by manual insertion may be explained by increased force when placing the manual IO needle. The complication may also be explained by lack of experience or unfamiliarity with the insertion device [5].

The complication rate with these devices also varies depending on the type of IO used. A bent catheter was the least common complication of the EZ-IO when compared to the other types of IO. Overall minimal complications were reported when using the semiautomatic intraosseous infusion system (EZ-IO) [23].

Uncommonly, life-threatening complications such as limb gangrene and compartment syndrome have been reported with this method of obtaining vascular access. One such event was reported by Greenstein et al. with extravasation of a vasopressor agent from the IO access leading to limb ischemia [8]. Other rare complications are reported such as bending of insertion needle, skin necrosis, retained needle end, and infection manifesting as osteomyelitis (Table 1) [2, 4–8].

### 3.4. Factors Determining Successful IO Placement in Adults

Singh et al. demonstrated that the success of IO placement, which was measured by rate of penetration of cortex at the first attempt, was around 66 percent [24]. Another study by Hafner et al. defined successful IO placement by meeting 2 of the 3 following criteria: aspirate bone marrow, infuse 10 mL methylene blue saline solution, and the absence of extravasation. 100 percent of the drill-assisted IO needles were successfully placed [5].

IO access can be difficult to obtain in obese patients. A prospective observational study was done on obese patients in which IO access was preceded by ultrasound guided measurement of soft tissue depth in accessible regions, that is, tibial tuberosity and proximal humerus. A higher BMI was found to be moderately predictive of an increased soft tissue depth at the proximal and distal tibia; however, this was not the case at the distal humerus. The size of the IO needle also determines the success of the procedure in obese patients. The standard IO needle measures 25 mm and is the adequate size for IO access in nonobese patients and for IO access at the proximal tibia and distal tibia if the patient's BMI is less than or equal to 43 and 60, respectively. It is advised that when attempting to gain intraosseous access at the humerus insertion site in an obese patient only a larger 45 mm needle should be used [25]. It has been shown time and again that training imparted to healthcare personnel can significantly improve the efficacy of intraosseous line placement [26]. Levitan et al. demonstrated that minimal training is required for the use of the EZ-IO device. In the study, the participants achieved insertion success after three attempts. The participants received one 5-minute in-service presentation and observed one insertion prior to their attempts [27].

## 4. Conclusion

Intraosseous access remains safe and easy to use if IV access is difficult and time consuming. There have been rare complications reported such as bending of insertion needle, skin necrosis, retained needle end, and infection manifesting as osteomyelitis [2, 4–8]. Evidence has shown that user training and the device used affect complication rates along with manual and semiautomatic insertion [5, 23]. Our reported case entails a semiautomatic insertion device with evidence of low complications, with limited user experience. Education should be used to facilitate learning experiences for all staff in the hospital for IO insertion. A suggested method of training for healthcare workers includes the initiation of a simulation training protocol for obtaining IO access. Implementation of a hospital wide training program would be relatively low cost and a low time burden. With education and training, EZ-IO may become the preferred method of achieving rapid vascular access for emergent resuscitation with a low risk for complications.

## Figures and Tables

**Table 1:** Complications associated with IO access.

Study	Type of study	Population studied	Number of IO placements	Number/percentage of major or minor complications	Number and type of major or minor complication
Hallas et al. (2013) [2]	Online questionnaire	Newborns to adults	861 (reporting EZ-IO only)	448/52%	25, extravasation 11, bent or broken needle 6, compartment syndrome
Lee et al. (2015) [4]	Prospective observational study	Unspecified, adults	33	3/9.09%	1, extravasation and skin necrosis 1, pain 1, dislodged needle
Hafner et al. (2013) [5]	Randomized prospective crossover experiment	Mixed breed swine	21	4/19%	3, unsuccessful infusion 1, extravasation
Paxton et al. (2009) [6]	Prospective cohort	Unspecified	30	17/57%	11, catheter dislodgement 3, inability to flush 2, failed attempt to place catheter 1, slow flow
Helm et al. (2015) [7]	Retrospective analysis	Newborns to adults	227	4/1.7%	2, needle dislocation 1, needle bending 1, extravasation

## References

[1] Orlowski J. P., Porembka D. T., Gallagher J. M., Lockrem J. D., VanLente F. (1990). Comparison study of intraosseous, central intravenous, and peripheral intravenous infusions of emergency drugs. *American Journal of Diseases of Children*.

[2] Hallas P., Brabrand M., Folkestad L. (2013). Complication with intraosseous access: Scandinavian users' experience. *Western Journal of Emergency Medicine*.

[3] Glaeser P. W., Hellmich T. R., Szewczuga D., Losek J. D., Smith D. S. (1993). Five-year experience in prehospital intraosseous infusions in children and adults. *Annals of Emergency Medicine*.

[4] Lee P. M. J., Lee C., Rattner P., Wu X., Gershengorn H., Acquah S. (2015). Intraosseous versus central venous catheter utilization and performance during inpatient medical emergencies. *Critical Care Medicine*.

[5] Hafner J. W., Bryant A., Huang F., Swisher K. (2013). Effectiveness of a drill-assisted intraosseous catheter versus manual intraosseous catheter by resident physicians in a swine model. *Western Journal of Emergency Medicine*.

[6] Paxton J. H., Knuth T. E., Klausner H. A. (2009). Proximal humerus intraosseous infusion: a preferred emergency venous access. *The Journal of trauma*.

[7] Helm M., Haunstein B., Schlechtriemen T., Ruppert M., Lampl L., Gäßler M. (2015). EZ-IO® intraosseous device implementation in German helicopter emergency medical service. *Resuscitation*.

[8] Greenstein Y. Y., Koenig S. J., Mayo P. H., Narasimhan M. (2016). A serious adult intraosseous catheter complication and review of the literature. *Critical Care Medicine*.

[9] Deakin C. D., Nolan J. P., Soar J. (2010). European resuscitation council guidelines for resuscitation 2010 section 4. Adult advanced life support. *Resuscitation*.

[10] Neumar R. W., Otto C. W., Link M. S. (2010). Part 8: adult advanced cardiovascular life support: 2010 American Heart Association guidelines for cardiopulmonary resuscitation and emergency cardiovascular care. *Circulation*.

[11] Lapostolle F., Catineau J., Garrigue B. (2007). Prospective evaluation of peripheral venous access difficulty in emergency care. *Intensive Care Medicine*.

[12] Zasko P., Szarpak L., Kurowski A., Truszewski Z., Czyzewski L. (2016). Success of intraosseous access procedure in simulated adult resuscitation. *Critical Care and Resuscitation*.

[13] Derikx H. J. G. M., Gerritse B. M., Gans R., van der Meer N. J. M. (2014). A randomized trial comparing two intraosseous access devices in intrahospital healthcare providers with a focus on retention of knowledge, skill, and self-efficacy. *European Journal of Trauma and Emergency Surgery*.

[14] Waisman M., Waisman D. (1997). Bone marrow infusion in adults. *Journal of Trauma-Injury, Infection and Critical Care*.

[15] Calkins M. D., Fitzgerald G., Bentley T. B., Burris D. (2000). Intraosseous infusion devices: a comparison for potential use in special operations. *Journal of Trauma-Injury, Infection and Critical Care*.

[16] Miller D. D., Guimond G., Hostler D. P., Platt T., Wang H. E. (2005). Feasibility of sternal intraosseous access by emergency medical technician students. *Prehospital Emergency Care*.

[17] Ong M. E. H., Chan Y. H., Oh J. J., Ngo A. S.-Y. (2009). An observational, prospective study comparing tibial and humeral intraosseous access using the EZ-IO. *The American Journal of Emergency Medicine*.

[18] Pasley J., Miller C. H. T., DuBose J. J. (2015). Intraosseous infusion rates under high pressure: a cadaveric comparison of anatomic sites. *Journal of Trauma and Acute Care Surgery*.

[19] Lairet J., Bebarta V., Lairet K. (2013). A comparison of proximal tibia, distal femur, and proximal humerus infusion rates using the EZ-IO intraosseous device on the adult swine (*Sus scrofa*) model. *Prehospital Emergency Care*.

[20] Warren D. W., Kissoon N., Sommerauer J. F., Rieder M. J. (1993). Comparison of fluid infusion rates among peripheral intravenous and humerus, femur, malleolus, and tibial intraosseous sites in normovolemic and hypovolemic piglets. *Annals of Emergency Medicine*.

[21] Internal study report, Protocol 2013-06: Clinical Studies to Determine the Optimal Technique to Identify the Proximal Humerus Intraosseous Vascular Access Insertion Site, Vidacare Corporation, May 2013

[22] Barlow B., Kuhn K. (2014). Orthopedic management of complications of using intraosseous catheters. *The American Journal of Orthopedics*.

[23] Brenner T., Bernhard M., Helm M. (2008). Comparison of two intraosseous infusion systems for adult emergency medical use. *Resuscitation*.

[24] Singh S., Aggarwal P., Lodha R. (2016). Feasibility study of a novel intraosseous device in adult human cadavers. *Indian Journal of Medical Research*.

[25] Kehrl T., Becker B. A., Simmons D. E., Broderick E. K., Jones R. A. (2016). Intraosseous access in the obese patient: assessing the need for extended needle length. *The American Journal of Emergency Medicine*.

[26] Cheung J., Rosenberg H., Vaillancourt C. (2014). Barriers and facilitators to intraosseous access in adult resuscitations when peripheral intravenous access is not achievable. *Academic Emergency Medicine*.

[27] Levitan R. M., Bortle C. D., Snyder T. A., Nitsch D. A., Pisaturo J. T., Butler K. H. (2009). Use of a battery-operated needle driver for intraosseous access by novice users: skill acquisition with cadavers. *Annals of Emergency Medicine*.

